# Risk analysis of fluctuating hypercalcemia after leukapheresis in cellular therapy

**DOI:** 10.1038/s41598-023-42159-1

**Published:** 2023-09-11

**Authors:** Tomoyasu Jo, Yasuyuki Arai, Toshio Kitawaki, Momoko Nishikori, Chisaki Mizumoto, Junya Kanda, Kouhei Yamashita, Miki Nagao, Akifumi Takaori-Kondo

**Affiliations:** 1 Center for Research and Application of Cellular Therapy, Kyoto University Hospital, 54 Shogoin Kawahara-cho, Sakyo-ku, Kyoto, 606-8507 Japan; 2 Department of Hematology and Oncology, Graduate School of Medicine, Kyoto University, Kyoto, Japan; 3 Department of Clinical Laboratory Medicine, Graduate School of Medicine, Kyoto University, Kyoto, Japan

**Keywords:** Outcomes research, Haematological cancer, Cancer immunotherapy

## Abstract

Optimized management of citrate-induced hypocalcemia is required to provide safe leukapheresis. We prospectively analyzed subjects who underwent leukapheresis for cytotherapy, and evaluated serum ionized (iCa) concentrations before, at the end of, and 1 h after leukapheresis. During leukapheresis, calcium gluconate solution was continuously supplemented intravenously with hourly measurement of iCa. 76 patients including 49 lymphapheresis for chimeric antigen receptor T-cell therapy and 27 stem cell collections were enrolled. Median processing blood volume was 10 L (range, 6–15 L). Fluctuating hypercalcemia, in which the iCa concentration rose above its upper limit 1 h after leukapheresis, was observed in 58 subjects (76.3%). Multivariate analysis revealed that higher ratios of processing blood volume to body weight, more rapid calcium supplementation, and lower iCa concentration at the end of leukapheresis significantly increased elevation of serum iCa concentration by 1 h after leukapheresis. Based on multivariate analyses, we developed a formula and a diagram that accurately estimates serum iCa concentration 1 h post-leukapheresis. This suggests optimal targets for iCa concentration and calcium supplementation rates. In cases with high ratios of processing blood volume to body weight, slowing the rate of blood processing, rather than increasing calcium supplementation should safely alleviate hypocalcemia during leukapheresis without inducing hypercalcemia thereafter.

## Introduction

The number of leukapheresis cases has been increasing with wider clinical application of cell therapy, including peripheral blood stem cell (PBSC) transplantation and chimeric antigen (CAR) T cell therapy^1, 2^. Thus, it is increasingly important to ensure the safety of healthy donors, as well as patients with poor general health conditions, who undergo leukapheresis.

Hypocalcemia is a well-known complication of leukapheresis, because citrate, which is used as an anticoagulant during leukapheresis, reversibly chelates ionized calcium (iCa) in the serum^3^. While calcium supplementation has been used to alleviate citrate-induced hypocalcemia, an optimal method of calcium supplementation has not been devised^4–6^. Moreover, because citrate is degraded promptly and bound calcium is released when citrate administration is terminated^3, 7^, inappropriate calcium supplementation can potentially cause a rapid rise in serum iCa after leukapheresis. Indeed, serious hypercalcemia associated with citrate anticoagulation has been reported in renal replacement therapy and apheresis^8–10^, and a healthy donor with transient impaired consciousness due to hypercalcemia after PBSC collection has been reported^11^. Therefore, it is essential to address fluctuation of serum iCa not only during leukapheresis, but also thereafter, in order to optimize calcium supplementation methods. However, information on this subject has been limited.

In this study, we (1) clarified changes in serum iCa concentration after leukapheresis performed for cellular therapy, (2) identified risk factors that lead to exacerbated changes in serum iCa after leukapheresis, and (3) devised an optimal calcium supplementation method that alleviates changes in iCa concentration. Our findings should help ensure safe leukapheresis for patients and healthy donors.

## Patients and methods

### Study cohort and inclusion criteria

This prospective study enrolled all consecutive leukapheresis subjects for autologous (auto) or allogeneic (allo) PBSC collection, and T-cell collection in CAR-T cell therapy performed for hematological disorders in adult patients at Kyoto University Hospital from April 2021 to August 2022. Those enrolled in clinical trials at the time of leukapheresis were excluded. This study was approved by the Institutional Review Board of Kyoto University and was conducted according to principles of the Declaration of Helsinki. Informed consent to be included in this study was obtained from all patients.

### Leukapheresis procedures

All leukapheresis procedures were performed using Spectra Optia Apheresis Systems (Terumo BCT, Tokyo, Japan) in either the MNC or CMNC program with acid citrate dextrose solution A (ACD-A) as an anticoagulant at a blood ratio of 12:1. Processing blood volumes were set according to ASBMT guidelines and recommendations for PBSC collection^12^, or guidelines provided by pharmaceuticals in leukapheresis for CAR-T cell therapy, basically with a maximum volume of 0.3 L/kg of body weight or 4 times the total blood volume (TBV). TBV of subjects was calculated using Nadler’s equation^13^. Estimated glomerular filtration rate (eGFR) was calculated using an equation from the Japanese Society of Nephrology which is based on the serum creatinine value^14^.

### Calcium supplementation

All subjects received 8.5% calcium gluconate solution (CALCICOL®, Nichi-iko, Toyama, Japan), which contains 78.5 mg (1.96 mmol) of calcium in 10 mL, by continuous intravenous (iv) infusion using syringe drivers during leukapheresis. The initial rate of 8.5% calcium gluconate solution administration was determined at the discretion of the attending physician (range, 10–20 mL/h). Thereafter, serum ionized calcium (iCa) concentration was measured hourly with a blood gas analysis system (RapidPoint 500®, Siemens Healthineers, Erlangen, Germany) using heparinized venous whole blood, and the rate of calcium gluconate administration was adjusted in the range of 10–25 mL/h, so that iCa concentration fell within the range of 3.0 to 5.0 mg/dL (0.75–1.25 mmol/L). When a patient had symptoms suggestive of hypocalcemia, such as tingling in the lips, fingers and feet, iCa concentration was additionally measured to adjust the rate of calcium gluconate administration.

### Measurement of iCa and tCa

**Figure 1:**
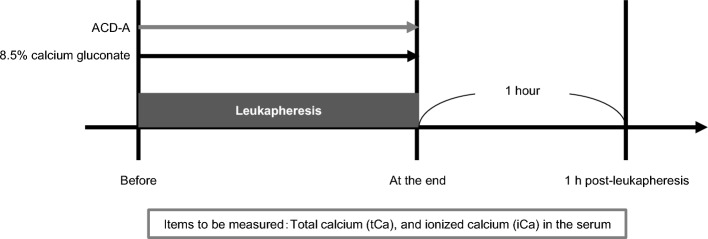
Study design. Serum total (tCa) and ionized calcium (iCa) concentrations were measured before, at the end of, and 1 h post-leukapheresis. As an anticoagulant, acid citrate dextrose solution A (ACD-A) was administered during leukapheresis. Continous intravenous administration of 8.5% calcium gluconate was performed during leukapheresis.

Serum iCa and tCa concentrations before, at the end of, and 1 h after apheresis were measured using serum centrifuged from venous whole blood (Fig. 1). iCa measurements were performed using an ion electrode method (SRL, Tokyo, Japan) at pH 7.40, 37 °C. Measurements of tCa were performed colorimetrically with an automatic analyzer LABOSPECT008α (Hitachi, Tokyo, Japan) using the reagent, Aqua-Auto Kainos Ca (Kainos Laboratories, Tokyo, Japan).

### Statistical analyses

For baseline patient characteristics, categorical variables were assessed using Fisher’s exact test, whereas continuous variables were compared using two-tailed unpaired Student’s t-tests, or one-way analysis of variance with Tukey’s post-hoc test. Continuous variables were summarized using medians and ranges, and categorical variables were summarized as counts and percentages. Predictive factors associated with the magnitude of the fluctuating increase in iCa were analyzed using Pearson’s correlation and univariate regression models. In multivariate analyses for prediction factors, all variables with p < 0.1 in univariate analyses were included and a multiple regression model with a backward stepwise elimination method (significance level = 0.05) was performed. In regression analyses, all continuous variables were log-transformed to normalize skewed distributions. All statistical analyses were performed using Stata software version 17 (Stata Corp., College Station, TX, USA).

## Results

### Subject and leukapheresis characteristics

Leukapheresis was performed on 76 subjects, including allo-PBSC in 6 subjects (7.9%), auto-PBSC in 21 subjects (27.6%), and CAR-T cell therapy in 49 subjects (64.5%). There were 47 males (61.8%) and 29 females (38.2%). Median age at leukapheresis was 61 years (range, 21–75 years) (Table 1). Median height, body weight, and total blood volume were 164.3 cm (range, 145.7–181.1 cm), 57.2 kg (range, 39.8–82.0 kg), and 41.2 dL (range, 27.4–51.4 dL), respectively. Laboratory peripheral blood data before leukapheresis were: hematocrit (hct), 30.1% (range, 23.9–46.1%), albumin (alb), 3.7 g/dL (range, 2.6–4.7 g/dL), total bilirubin (T-Bil), 0.5 mg/dL (range, 0.3–2.3 mg/dL), and eGFR, 74.3 mL/min/1.73 m^2^ (range, 27.4–176.1 mL/min/1.73 m^2^). No subject had significantly impaired liver function. For leukapheresis, a median of 10 L (range, 6–15 L) of blood was processed with a median processing time of 219 min (range, 110–328 min). The median volume of ACD-A administered during leukapheresis was 916 mL (range, 546–1364 mL), and the median volume of 8.5% calcium gluconate supplemented was 55 mL (range, 24–80 mL), which contained 432 mg (range, 188–628 mg) of calcium. While allo-PBSC donors were younger and heavier than auto-PBSC and CAR-T patients, processed blood volume, processed-blood-volume-to-total-blood-volume ratios, dose of ACD-A solution, and dose of 8.5% calcium gluconate solution were comparable between groups (Supplementary Table S1).

**Table 1:** Subject and leukapheresis characteristics.

	Total
	N = 76
Patient background	
Age (year)	61 (21–75)
Sex	
Male	47 (61.8%)
Female	29 (38.2%)
Height (cm)	164.3 (145.7–181.1)
Body weight (kg)	57.2 (39.8–82.0)
Total blood volume (Nadler) (dL)	41.2 (27.4–51.4)
Type of apheresis	
Allo-PBSC	6 (7.9%)
Auto-PBSC	21 (27.6%)
CAR-T	49 (64.5%)
Laboratory data before leukapheresis	
Hct (%)	30.1 (23.9–46.1)
Alb (g/dL)	3.7 (2.6–4.7)
T-Bil (mg/dL)	0.5 (0.3–2.3)
eGFR (mL/min/1.73m^2^)	74.3 (27.4–176.1)
Leukapheresis parameters	
Blood volume processed (L)	10.0 (6.0–15.0)
Duration of apheresis (min)	219 (110–328)
Dos of ACD-A (mL)	916 (546–1364)
Dose of 8.5% calcium gluconate (mL)	55 (24–80)

ACD-A, acid citrate dextrose solution A; Alb, albumin; allo-PBSC, allogeneic peripheral blood stem cell harvest; auto-PBSC, autologous peripheral blood stem cell harvest; CAR-T, chimeric antigen receptor T-cell therapy; eGFR, estimated glomerular filtration rate; Hct, hematocrit; iCa, ionized calcium; T-Bil, total bilirubin; TBV, total blood volume; tCa, total calcium. Continuous variables were summarized using medians and ranges, and categorical variables were summarized as counts and percentages. Normal ranges of laboratory values: Hct, 40.7–50.1% for male, 35.1–44.4% for female; Alb, 4.1–5.1 g/dL; T-Bil, 0.4–1.5 mg/dL; eGFR, ≥ 90 ml/min/1.73 m^2^.

### Kinetics of serum ionized and total calcium concentrations

**Figure 2:**
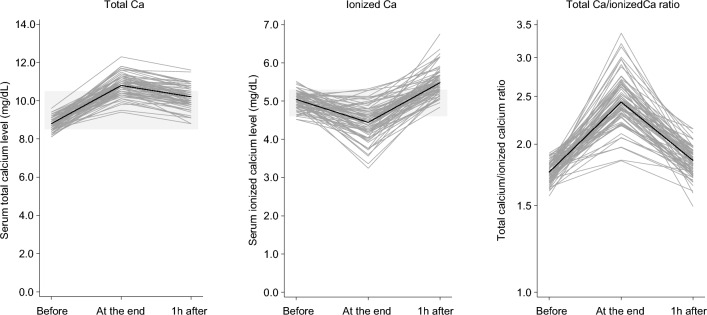
Kinetics of serum total (tCa) and ionized calcium (iCa) concentrations during peri-apheresis periods. Changes in tCa concentration (left panel), iCa concentration (center panel), and tCa/iCa ratios (right panel) are shown. Bold lines indicate fluctuation of median values. Gray stripes indicate ranges of normal values (tCa, 8.5–10.5 mg/dL; iCa, 4.6–5.3 mg/dL). The axis for tCa/iCa ratio is logarithmic.

In this study, serum tCa and iCa concentrations were measured before, at the end of, and 1 h post-leukapheresis (Fig. 1). Before leukapheresis, median serum tCa and iCa concentrations were 8.8 mg/dL (range, 8.1–9.6 mg/dL) and 5.04 mg/dL (range, 4.52–5.52 mg/dL) (Table 2). While calcium was supplemented with continuous 8.5% calcium gluconate infusion during leukapheresis, serum iCa concentration decreased from the start to the end of leukapheresis in 68 subjects (89.4%), and 48 subjects (63.2%) were below the lower limit of normal (4.6 mg/dL) at the end of leukapheresis (Fig. 2). On the other hand, tCa concentration increased during leukapheresis. Median serum tCa and iCa concentrations, and resultant tCa/iCa ratios at the end of leukapheresis were 10.8 mg/dL (range, 9.4–12.3 mg/dL) and 4.44 mg/dL (range, 3.24–5.32 mg/dL), and 2.43 (range, 1.85–3.36), respectively. Then, after leukapheresis, while serum tCa concentration decreased only slightly, the tCa/iCa ratio returned to baseline and there was a sharp rise in serum iCa concentration (Fig. 2). The type of leukapheresis did not significantly affect increased iCa or tCa concentration (Supplementary Fig. S1 and Supplementary Table S2). In 58 subjects (76.3%), 1 h post-leukapheresis, iCa concentration was above the upper limit of normal. In 33 subjects (43.3%), iCa concentration was below the lower limit of normal range at the end of leukapheresis, but rose above the upper limit of the normal range 1 h after the end of leukapheresis, with a prominent rise during the first hour after the end. These results suggest that hypercalcemia occurs after leukapheresis in the majority of subjects.

**Table 2:** Changes in serum pH, ionized and total calcium concentrations during the peri-apheresis period.

	Total
	N = 76
Before leukapheresis	
pH	7.37 (7.32–7.43)
iCa (md/dL)	5.04 (4.52–5.52)
tCa (md/dL)	8.8 (8.1–9.6)
At the end of leukapheresis	
pH	7.44 (7.36–7.50)
iCa (md/dL)	4.44 (3.24–5.32)
tCa (md/dL)	10.8 (9.4–12.3)
1 h post-leukapheresis	
iCa (md/dL)	5.48 (4.84–6.76)
tCa (md/dL)	10.2 (8.8–11.6)

iCa, ionized calcium; tCa, total calcium. Continuous variables were summarized using medians and ranges. Normal ranges of laboratory values: pH, 7.35–7.45; iCa, 4.6–5.3 mg/dL; tCa 8.5–10.5 mg/dL.

### Factors associated with fluctuating elevation of serum ionized calcium concentration after leukapheresis

**Figure 3:**
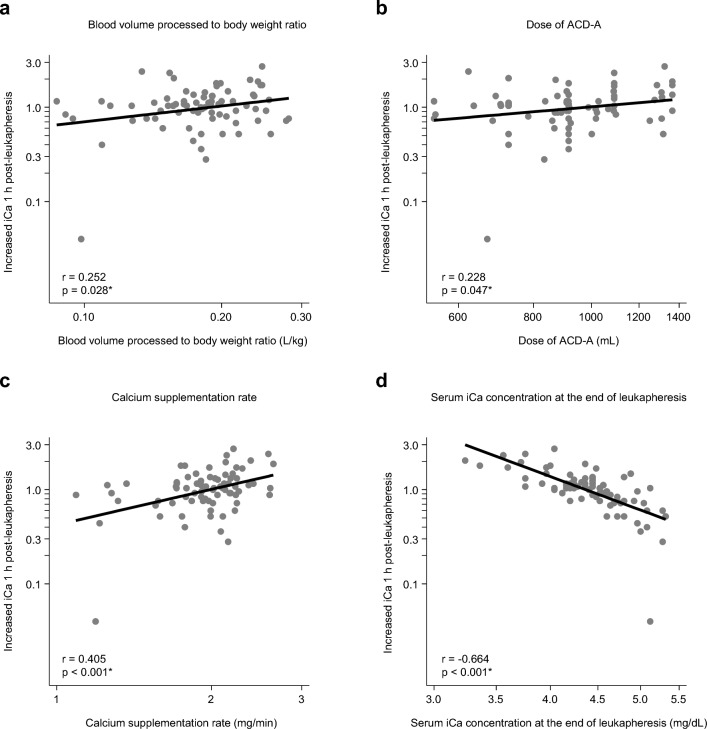
Correlation between increased serum ionized calcium (iCa) 1 h post-leukapheresis and leukapheresis parameters. (**a**) The ratio of blood volume processed to body weight. (**b**) Acid citrate dextrose (ACD-A) dose. (**c**) Calcium supplementation rate. (**d**) Serum iCa concentration at the end of leukapheresis. Axes are logarithmic. *Indicates p < 0.05.

To investigate factors associated with the degree of fluctuating serum iCa after leukapheresis, we evaluated correlations of various clinical factors and laboratory data with increased serum iCa concentration from the end to 1 h post-leukapheresis. In univariate analysis, pre-leukapheresis background factors, such as age, sex, body weight, height, and total blood volume at leukapheresis, did not correlate significantly with increased serum iCa concentration (Table 3, and Supplementary Fig. S2). While this study included subjects with reduced eGFR associated with old age, eGFR did not correlate significantly with increased serum iCa concentration. For leukapheresis factors, there were significant positive correlations between increased iCa concentration and the ratio of processed blood volume to body weight, volume of ACD-A administered during leukapheresis, calcium supplementation rate, and serum tCa concentration at the end of leukapheresis, whereas serum iCa concentration at the end of leukapheresis was negatively and significantly correlated with increased iCa concentration (Table 3 and Fig. 3).

**Table 3:** Univariate and multivariate analyses of clinical parameters for increased ionized calcium concentration.

		Univariate				Multivariate			
		Coefficient	LO_95%CI	UP_95%CI	p value	Coefficient	LO_95%CI	UP_95%CI	p value
Patient background									
Age	/year	0.314	− 0.069	0.697	0.107				
Sex	Female vs. male	0.196	− 0.070	0.462	0.146				
Height	/cm	−2.039	− 4.899	0.821	0.160				
Body weight	/kg	−0.448	− 1.317	0.420	0.307				
TBV	/dL	−0.598	− 1.418	0.223	0.151				
Type of leukapheresis	Auto-PB vs. allo-PB	0.240	− 0.287	0.768	0.366				
	CAR-T vs. allo-PB	0.298	− 0.195	0.790	0.232				
Laboratory baseline									
Hct	/%	−0.081	− 0.817	0.654	0.826				
Alb	/g/dL	−0.249	− 1.435	0.938	0.677				
T-Bil	/mg/dL	−0.126	− 0.435	0.183	0.420				
eGFR	/mL/min/1.73 m^2^	−0.348	− 0.822	0.126	0.148				
pH		26.656	− 14.018	67.330	0.196				
iCa	/mg/dL	0.990	− 1.881	3.861	0.494				
tCa	/mg/dL	−0.080	− 3.605	3.445	0.964				
Leukapheresis parameters									
Blood volume processed/body weight	/L/kg	0.553	0.060	1.047	0.028*	0.425	0.057	0.793	0.024*
Duration of leukapheresis	/min	−0.340	− 0.881	0.201	0.214				
Dose of ACD-A	/mL	0.548	0.007	1.089	0.047*				
Calcium supplementation rate	/mg/min	1.254	0.598	1.909	< 0.001*	0.586	0.048	1.123	0.033*
Laboratory at the end of leukapheresis									
pH		4.195	− 29.392	37.783	0.804				
iCa	/mg/dL	−3.674	− 4.631	− 2.716	< 0.001*	− 3.383	− 4.307	− 2.458	< 0.001*
tCa	/mg/dL	4.054	1.575	6.532	0.002*				

Abbreviations and normal ranges of laboratory values are shown in Tables 1 and 2. *Indicates p < 0.05.

Multivariate regression analysis estimated that the following three factors were independently associated with increased serum iCa concentration from the end of to 1 h post-leukapheresis: a higher ratio of processed blood volume to body weight (coefficient, 0.425 per L/kg; 95% confidence interval [CI], 0.057–0.793; p = 0.024), a higher calcium supplementation rate during leukapheresis (coefficient, 0.586 per mg/min; 95% CI, 0.048–1.123; p = 0.033), and a lower serum iCa concentration at the end of leukapheresis (coefficient, − 3.383 per mg/dL; 95% CI, − 4.307 to − 2.458; p < 0.001) (Table 3).

### Predictive model for fluctuating elevation of ionized calcium concentration

On the basis of the multivariate regression model (Table 3), the estimated increase in serum iCa concentration from the end of leukapheresis to 1 h post-leukapheresis was calculated using the formula (see also Supplementary Fig. S3): Estimated increase in serum iCa concentration = 207.096 × (blood volume processed [L]/body weight [kg])^0.425^ × (calcium supplementation rate [mg/min])^0.586^ × (serum iCa concentration at the end of leukapheresis [mg/dL])^–3.383^.

**Figure 4:**
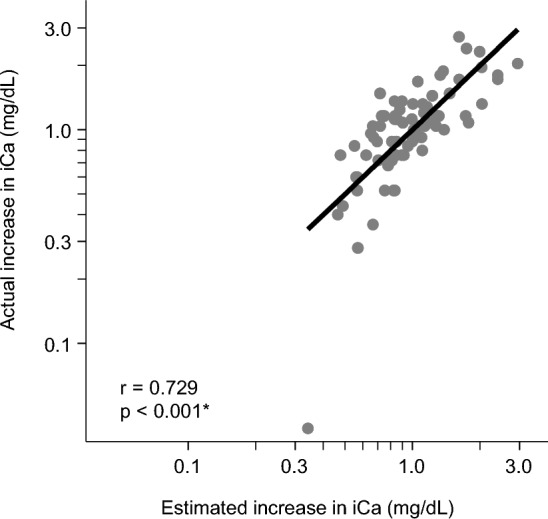
Correlation between actual and estimated increase in serum ionized calcium (iCa) concentration 1 h post-leukapheresis. Axes are logarithmic. *Indicates p < 0.05.

The estimated increase in serum iCa concentration correlated significantly with the actual increase in serum iCa concentration from the end of to 1 h post-leukapheresis (r = 0.729, p < 0.001) (Fig. 4), suggesting that our estimation equation is suitable to predict elevation of serum iCa concentration after leukapheresis.

**Figure 5:**
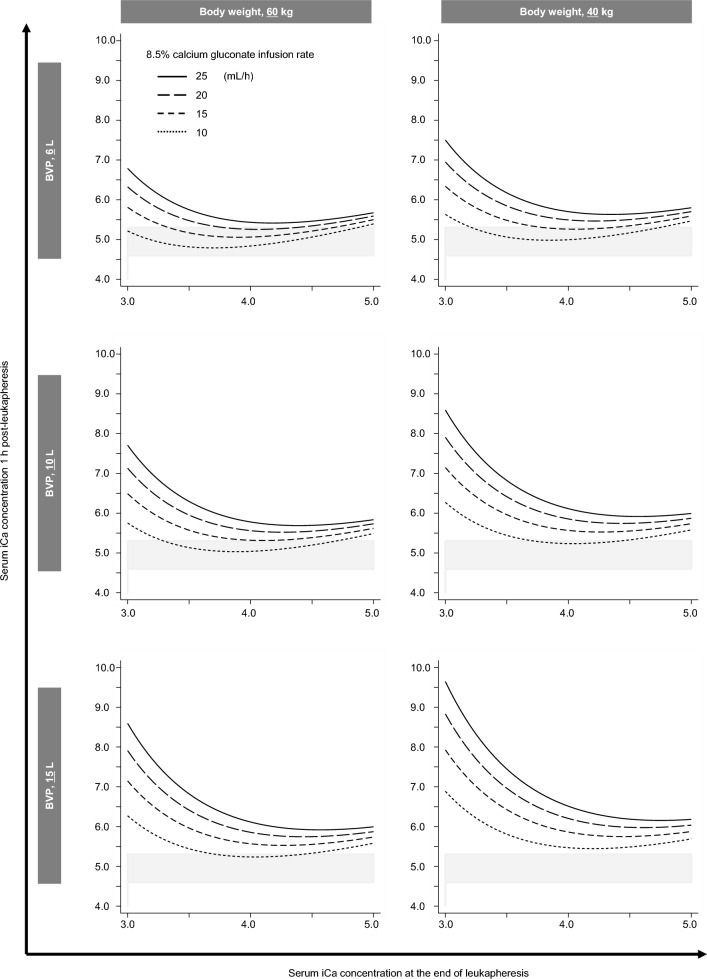
Quick reference diagrams estimating serum ionized calcium (iCa) concentration 1 h post-leukapheresis. Serum iCa concentration at the end of leukapheresis and serum iCa concentration 1 h post-leukapheresis are described as per blood volume processed (BVP) and body weight. Solid, long dashed, short dashed, and dotted lines indicate 25, 20, 15, and 10 mL/h of 8.5% calcium gluconate infusion rate. Gray stripes indicate the range of normal values of serum iCa (4.6–5.3 mg/dL).

Then, in order to optimize management of hypocalcemia related to citrate containing ACD-A infusion, quick reference diagrams based upon the calcium supplementation rate during leukapheresis (converted to infusion rate of 8.5% calcium gluconate solution that contain 7.85 mg calcium per mL), and serum iCa concentration at the end of leukapheresis, that show serum iCa concentration 1 h post-leukapheresis, were developed according to each clinical setting (Fig. 5). We assumed several clinical situations as per combination of blood volume processed, and body weight. In each situation, the relationship between serum iCa concentration at the end of leukapheresis, and serum iCa concentration 1 h post-leukapheresis is shown, where line types indicate calcium supplementation rate. These plots pose importance of serum iCa concentration at the end of leukapheresis and calcium supplementation rate to serum iCa concentration 1 h post-leukapheresis. For instance, in a subject weighing 60 kg who underwent leukapheresis with 6 L of blood volume processed, the diagram (top left panel in Fig. 5) shows clearly that the serum iCa concentration 1 h post-leukapheresis differs widely depending both on the serum iCa concentration at the end of leukapheresis and calcium supplementation rate. In this case, when serum iCa concentration at the end of leukapheresis is 4.0 mg/dL, serum iCa concentration 1 h post-leukapheresis corresponding to 8.5% calcium gluconate supplementation rates of 10, and 25 mL/h are 4.8 and 5.4 mg/dL, and when serum iCa concentration at the end of leukapheresis is 3.0 mg/dL, those corresponding to 8.5% calcium gluconate supplementation rate of 10, and 25 mL/h are 5.2, and 6.8 mg/dL, respectively. These results suggest that hypercalcemia after leukapheresis is more pronounced in subjects in whom iCa concentrations at the end of leukapheresis are low, especially below 3.5 mg/mL, despite a faster rate of calcium supplementation. Moreover, the marked fluctuating elevation seen with low iCa concentration at the end of leukapheresis despite sufficient calcium supplementation was more prominent in subjects with low body weight for whom a large blood volume was processed during leukapheresis (bottom right panel in Fig. 5).

## Discussion

In this prospective study, we evaluated 76 subjects who received leukapheresis for cell therapy, including CAR-T cell therapy and stem cell transplantation, and found the following: (1) Fluctuating elevation of serum iCa concentration occurred after leukapheresis. (2) The degree of elevation of serum iCa concentration after leukapheresis was significantly enhanced by the following three factors: a higher ratio of blood volume processed per unit of body weight, a faster rate of calcium supplementation during leukapheresis, and a lower concentration of serum iCa at the end of leukapheresis. (3) A novel model was developed to estimate serum iCa concentration 1 h post-leukapheresis based upon subject and leukapheresis parameters.

First, we observed that serum iCa concentration exceeded the upper normal range 1 h post-leukapheresis in many subjects (76.3%), even if it was within the normal range or below the lower limit of the normal range at the end of leukapheresis. Because hypocalcemia induced by citrate chelation of calcium can sometimes cause serious arrhythmias, calcium supplementation is used during leukapheresis to alleviate hypocalcemia. However, citrate is degraded with a half-life as short as 30 min^7^, whereas calcium regulation in the body through renal excretion and bone resorption is relatively slow^15, 16^. Thus, when citrate administration is terminated at the end of leukapheresis, calcium conjugated by citrate is released, resulting in an increase in iCa. Actually, there are several reports of impaired consciousness due to hypercalcemia after leukapheresis^11^. Therefore, it is necessary to investigate factors influencing elevation of serum iCa concentration after leukapheresis to prevent hypercalcemia. Given the lack of parallel kinetics of iCa and tCa concentrations along with the prominent change in iCa/tCa ratios observed in this study (Fig. 2), it is important to measure both iCa and tCa concentrations during the peri-apheresis period for accurate evaluation of calcium.

In this study, multivariate analysis identified a higher ratio of blood volume processed per body weight, a faster rate of calcium supplementation, and a lower iCa concentration at the end of apheresis as risk factors for a greater fluctuation of iCa after leukapheresis. The amount of citrate administered during leukapheresis increases in proportion to the amount of blood volume processed. Because, in this study, to achieve a target cell number, we set a sufficient processing blood volume within the upper limit (determined from body weight or TBV), a smaller body weight, or TBV, results in a relatively larger increase in blood volume processed per body weight. Also, as citrate is metabolized in skeletal muscle, liver and kidney, a smaller body weight is associated with a slower citrate metabolism and leads to accumulation of citrate in the body during leukapheresis^4, 17, 18^. Thus, in cases with high processing blood volume and low body weight, the amount of citrate-bound calcium is larger during leukapheresis, and once leukapheresis is completed, citrate is degraded and free calcium is increased, resulting in elevation of iCa.

In the current study, a quick measurement of iCa with a blood gas analyzer was performed every hour during leukapheresis to adjust the rate of calcium supplementation. Thus, low iCa at the end of leukapheresis and a fast rate of calcium supplementation translates into reduced iCa despite sufficient calcium supplementation during leukapheresis due to citrate accumulation during apheresis^19^, which in turn leads to an elevation of iCa as a result of citrate degradation after apheresis. Therefore, low serum iCa during leukapheresis and/or an increased rate of calcium supplementation required to maintain ionized calcium can be used as indicators of citrate accumulation and rebound elevation of serum iCa thereafter.

Next, based on multivariate regression analyses, we developed a predictive formula for elevation of serum iCa concentration after leukapheresis (Fig. 4). In order to apply the formula in clinical practice without a need for complex calculations, we also developed a diagram that estimates serum iCa concentration 1 h post-leukapheresis, depending on leukapheresis parameters (Fig. 5). Notably, this diagram clarifies the importance of serum iCa concentration at the end of leukapheresis and the rate of calcium supplementation. As the rate of calcium supplementation and the target iCa concentration at the end of leukapheresis are modifiable, calcium management during leukapheresis can be optimized using this diagram. This diagram suggests that in a case with a high ratio of blood volume processed to body weight, it would be better to maintain serum iCa concentration during leukapheresis by reducing the rate of blood processing and the influx of citrate containing ACD-A solution, rather than by increasing the infusion rate of calcium supplementation.

While the strength of this study includes detailed analyses using real-world data, including subjects with various characteristics, there are limitations of the study. First, as calcium was continuously supplemented *i.v.* with hourly measurement of iCa during leukapheresis in this study, the validity of this study should be evaluated in leukapheresis using other calcium supplementation methods. Second, as both development and validation of the estimation model were performed using the same cohort, the potential for overfitting cannot be excluded. Therefore, despite suitable estimation accuracy, our model requires external validation to ensure generalizability, as well as further refinement in predictive performance. Third, endocrine factors, such as the parathyroid hormone/calcitonin system, which can influence calcium regulation were not analyzed in this study, although it is presumed that they have a minor influence during the short period of time investigated in this study. Fourth, respiratory factors that can affect acid–base balance were not analyzed in this study, and no episodes of overt hyperventilation or hypoventilation were recorded in these subjects. Fifth, correlation between the extent of post-leukapheresis hypercalcemia and clinical symptoms was not clarified in this study. While several patients in this cohort had anorexia after leukapheresis, its association with hypercalcemia was not clear, since other factors, such as G-CSF administration or admission-related environmental change, could modify symptoms. Since patients may present hypercalcemia-related symptoms after leukapheresis after leaving the apheresis unit, it is important to share results of this study with all departments of the apheresis facility involved in leukapheresis, and hypercalcemia-related symptoms should be further assessed in future.

In conclusion, the present study provides information regarding changes in serum iCa concentration, especially post-leukapheresis for cell therapy. We believe that these study results and discussion will help prevent fluctuating hypercalcemia after leukapheresis and will enable safer leukapheresis procedures for cell therapy in a wider range of donors and patients.

### Supplementary Information

Supplementary Information.

## Data Availability

Datasets used and/or analyzed during the current study are available from the corresponding author on reasonable request.
