# Supplementary material for: Risk analysis of fluctuating hypercalcemia after leukapheresis in cellular therapy
Source: Sci Rep. 2023 Sep 11;13:14952. doi: 10.1038/s41598-023-42159-1 (PMC10495341; doi:10.1038/s41598-023-42159-1)
Supplement: Supplementary file 1 — Supplementary Information. [file 41598_2023_42159_MOESM1_ESM.pdf]

Supplementary Information

Risk analysis of fluctuating hypercalcemia after leukapheresis in cellular therapy

Tomoyasu Jo, Yasuyuki Arai, Toshio Kitawaki, Momoko Nishikori, Chisaki Mizumoto, Junya Kanda, Kouhei Yamashita, Miki Nagao, and Akifumi Takaori-Kondo

Supplementary Table S1. Subject and leukapheresis characteristics according to type of leukapheresis

	Allo-PBSC N = 6	Auto-PBSC N = 21	CAR-T N = 49	p-value
**Patient background**				
Age (year)	21 (21-43)	63 (49-68)	62 (23-75)	<0.001*
Sex				0.147
Male	6 (100.0%)	12 (57.1%)	29 (59.2%)	
Female	0 (0.0%)	9 (42.9%)	20 (40.8%)	
Height (cm)	173.9 (159.2-174.2)	162.0 (153.9-170.1)	164.4 (145.7-181.1)	0.025*
Body weight (kg)	60.6 (57.7-69.9)	55.5 (46.5-67.3)	56.2 (39.8-82.0)	0.471
Total blood volume (Nadler) (dL)	44.4 (41.1-47.9)	38.8 (32.9-45.1)	41.5 (27.4-51.4)	0.072
**Laboratory before apheresis**				
Hct (%)	43.7 (41.1-46.1)	29.9 (23.9-38.5)	29.6 (24.0-45.5)	<0.001*
Alb (g/dL)	4.3 (4.0-4.4)	3.5 (2.8-3.9)	3.8 (2.6-4.7)	<0.001*
T-Bil (mg/dL)	0.7 (0.4-1.4)	0.4 (0.3-1.6)	0.5 (0.3-2.3)	0.337
eGFR (mL/min/1.73m^2)	92.0 (79.8-95.5)	78.7 (44.9-115.3)	72.6 (27.4-176.1)	0.289
**Apheresis parameters**				
Blood volume processed (L)	10.0 (8.3-12.0)	10.0 (7.0-10.0)	10.0 (6.0-15.0)	0.498
Blood volume processed (L)/TBV (L) ratio	2.3 (1.9-2.9)	2.5 (2.1-3.0)	2.9 (1.3-4.5)	0.256
Duration of apheresis (min)	287 (234-328)	221 (152-287)	205 (110-297)	0.002*
Dose of ACD-A (mL)	967 (916-1098)	916 (623-1308)	909 (546-1364)	0.805
Dose of 8.5% calcium gluconate (mL)	50 (40-58)	56 (50-70)	50 (24-80)	0.319

Abbreviations: ACD-A, acid citrate dextrose solution A; Alb, albumin; allo-PBSC, allogeneic peripheral blood stem cell harvest; auto-PBSC, autologous peripheral blood stem cell harvest; CAR-T, chimeric antigen receptor T-cell therapy; eGFR, estimated glomerular filtration rate; Hct, hematocrit; iCa, ionized calcium; T-Bil, total bilirubin; TBV, total blood volume; tCa, total calcium. Continuous variables were summarized using medians and ranges, and categorical variables were summarized as counts and percentages. Normal ranges of laboratory values: Hct, 40.7–50.1% for male, 35.1–44.4% for female; Alb, 4.1–5.1 g/dL; T-Bil, 0.4–1.5 mg/dL; eGFR, ≥90 ml/min/1.73m^2.

Supplementary Table S2. Changes in serum pH, ionized and total calcium concentrations during peri-apheresis period according to type of leukapheresis

	Allo-PBSC N = 6	Auto-PBSC N = 21	CAR-T N = 49	p-value
Before leukapheresis				
pH	7.36 (7.34-7.39)	7.37 (7.32-7.42)	7.36 (7.32-7.43)	0.519
iCa (md/dL)	5.00 (4.64-5.36)	4.96 (4.52-5.52)	5.12 (4.52-5.48)	0.090
tCa (md/dL)	9.1 (8.8-9.3)	8.7 (8.1-9.4)	8.9 (8.2-9.6)	0.059
At the endo of leukapheresis				
pH	7.42 (7.40-7.45)	7.45 (7.39-7.48)	7.44 (7.36-7.50)	0.033*
iCa (md/dL)	4.64 (4.44-4.96)	4.44 (3.36-5.28)	4.44 (3.24-5.32)	0.357
tCa (md/dL)	10.8 (10.3-10.9)	11.0 (10.4-11.8)	10.8 (9.4-12.3)	0.179
1 h after the end of leukaapheresis				
iCa (md/dL)	5.44 (5.32-6.76)	5.44 (4.84-6.16)	5.52 (4.96-6.76)	0.262
tCa (md/dL)	10.1 (9.5-10.5)	10.3 (9.7-11.0)	10.0 (8.8-11.6)	0.147

Abbreviations: iCa, ionized calcium; tCa, total calcium. Continuous variables were summarized using medians and ranges. Normal range of laboratory values: pH, 7.35–7.45; iCa, 4.6–5.3 mg/dL; tCa 8.5–10.5 mg/dL.

Supplementary Figure S1

Kinetics of serum iCa concentration according to type of leukapheresis

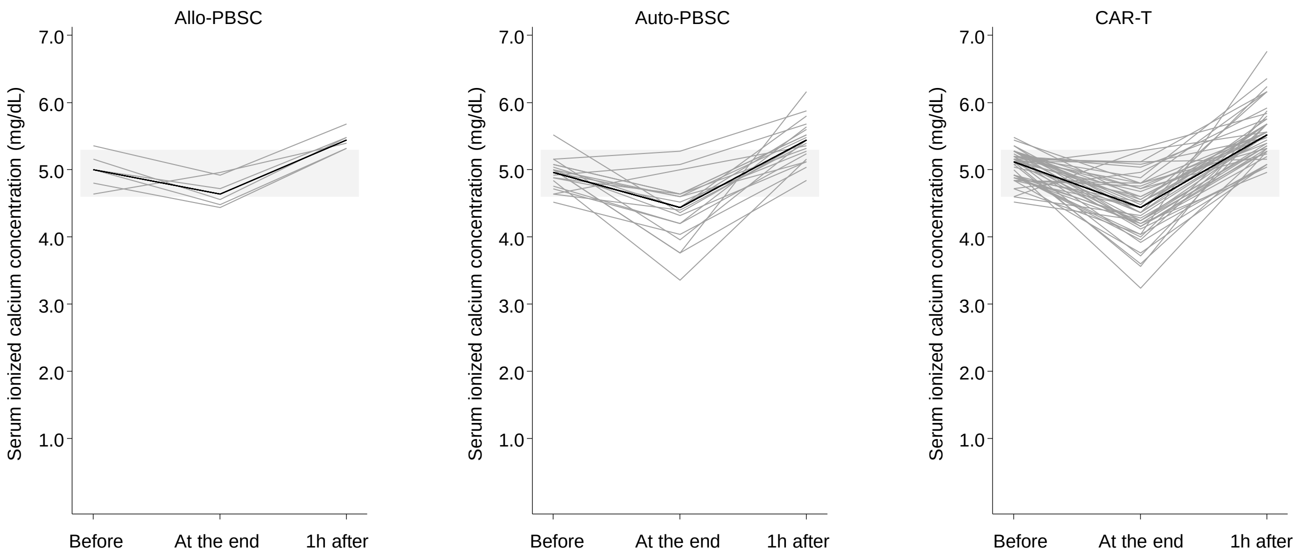

Supplementary Figure S1. Kinetics of serum ionized calcium (iCa) concentration during the peri-apheresis periods according to type of leukapheresis. Changes in iCa concentration according to type of apheresis: allogeneic peripheral blood stem cell (allo-PBSC) harvest (left panel); autologous peripheral blood stem cell (auto-PBSC) harvest (center panel), and lymphapheresis for chimeric antigen receptor T (CAR-T) cell therapy. Bold lines indicate fluctuation of median values. Gray stripes indicate the range of normal values (iCa, 4.6–5.3 mg/dL).

Supplementary Figure S2

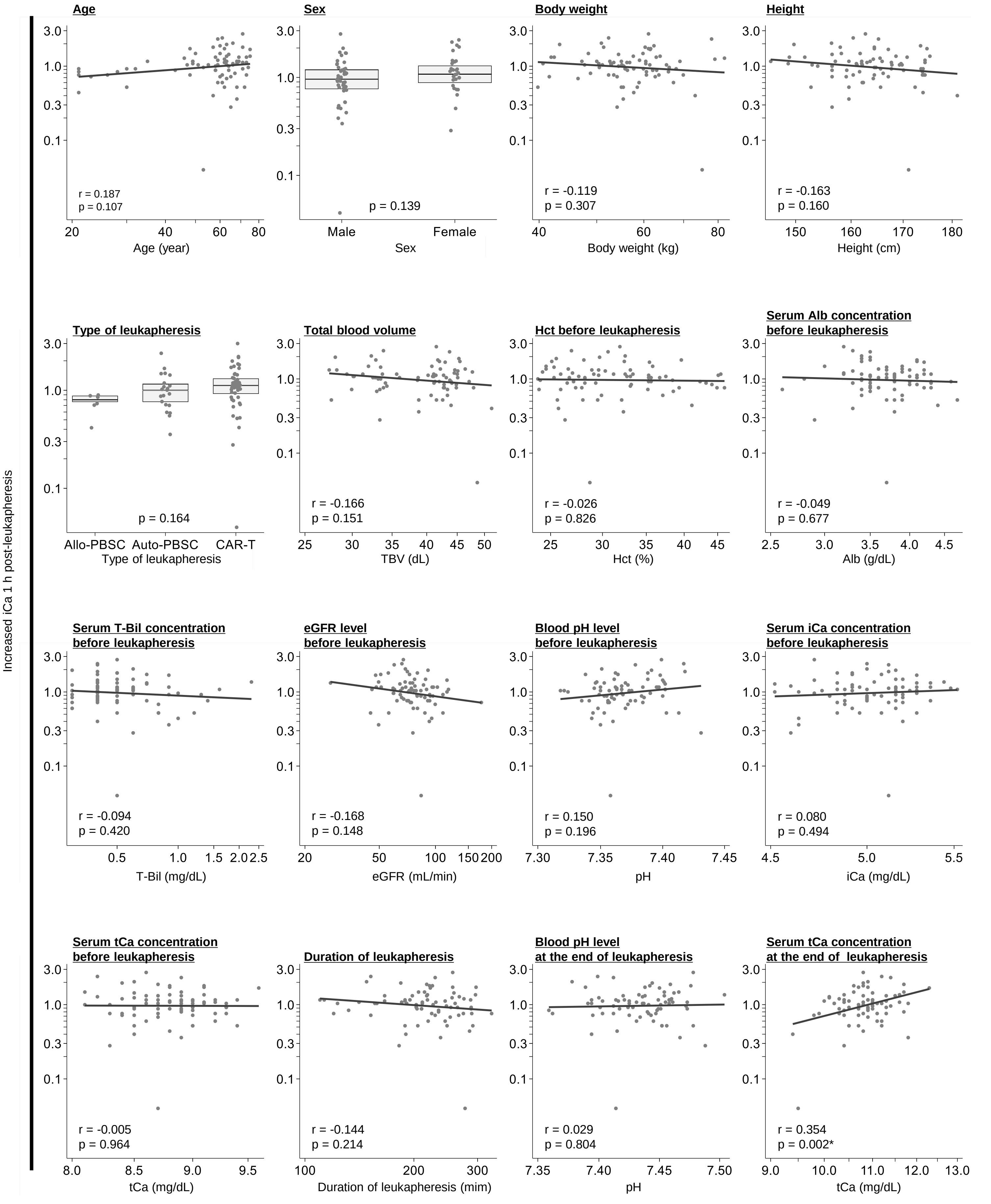

Supplementary Figure S2. Correlation between increased serum ionized calcium (iCa) concentration 1 h post-leukapheresis and clinical parameters.

Abbreviations: Alb, albumin; allo-PBSC, allogeneic peripheral blood stem cell harvest; auto-PBSC, autologous peripheral blood stem cell harvest; CAR-T, chimeric antigen receptor T-cell therapy; eGFR, estimated glomerular filtration rate; Hct, hematocrit; T-Bil, total bilirubin; TBV, total blood volume; tCa, total calcium. * indicates $p < 0.05$.

Supplementary Figure S3

$$\log(y) = a1 \times \log(x1) + a2 \times \log(x2) + \cdots \ldots + b$$

$$\therefore \mathrm{y} = e^{\log(y)} = e^{(a1 \times lox(x1) + \cdots + b)}$$

$$\therefore y = x1^{a1} \times x2^{a2} \times \cdots \times e^{b}$$

where
x1, x2, …: continuous variables
(for example, ratio of blood volume processed to body weight, and calcium supplementation rate)
a1, a2, …: coefficients for *x1, x2,* …
b: intercept
y: estimated increase in serum ionized calcium (iCa) concentration 1 h post-leukapheresis (mg/dL)

Supplemental Figure S3. Formula for estimated increased serum ionized calcium (iCa) concentration 1 h post-leukapheresis based on multivariate model.
